# Exploring genetic alterations in circulating tumor DNA from cerebrospinal fluid of pediatric medulloblastoma

**DOI:** 10.1038/s41598-021-85178-6

**Published:** 2021-03-11

**Authors:** Yanling Sun, Miao Li, Siqi Ren, Yan Liu, Jin Zhang, Shuting Li, Wenchao Gao, Xiaojun Gong, Jingjing Liu, Yuan Wang, Shuxu Du, Liming Sun, Wanshui Wu, Yongji Tian

**Affiliations:** 1 Department of Pediatrics, Beijing Shijitan Hospital, Capital Medical University, 10E. Tieyi Road, Yangfangdian, Haidian District, Beijing, China; 2 Department of Pediatric Neurosurgery, Beijing Tiantan Hospital, Capital Medical University, 119E. South Fourth Ring West Road, Fengtai District, Beijing, China

**Keywords:** Cancer genomics, CNS cancer, Paediatric cancer

## Abstract

Medulloblastoma (MB) is the most common type of brain malignancy in children. Molecular profiling has become an important component to select patients for therapeutic approaches, allowing for personalized therapy. In this study, we successfully identified detectable levels of tumor-derived cell-free DNA (cfDNA) in cerebrospinal fluid (CSF) samples of patients with MB. Furthermore, cfDNA from CSF can interrogate for tumor-associated molecular clues. MB-associated alterations from CSF, tumor, and post-chemotherapy plasma were compared by deep sequencing on next-generation sequencing platform. Shared alterations exist between CSF and matched tumor tissues. More alternations were detected in circulating tumor DNA from CSF than those in genomic DNA from primary tumor. It was feasible to detect MB-associated mutations in plasma of patients treated with chemotherapy. Collectively, CSF supernatant can be used to monitor genomic alterations, as a superior technique as long as tumor-derived cfDNA can be isolated from CSF successfully.

## Introduction

Medulloblastoma (MB) is the most common primary malignant tumor in children, accounting for 20% of all pediatric intracranial tumors^1^. Currently, MB is recognized as an umbrella term that encompasses various molecular pathological entities. Based on 2016 World Health Organization (WHO) classification of central nervous system (CNS) tumors, MB was divided into four subgroups, i.e., WNT, SHH, Group 3 and Group 4^2^. About one-third of children with MB have cerebrospinal fluid, cranial or spinal metastases at diagnosis^3^. Patients who suffer from recurrence or tumor progression, even with advanced treatment protocols, have dismal prognosis. Nowadays, cancer genome landscape is essential for diagnosis, prognostic evaluation and treatment selection. Unfortunately, for patients with tumor progression or recurrence, it is difficult to detect molecular alterations, because of unobtainable tumor tissue and lack of biopsy sample. Only advanced imaging technologies, such as MRI, CT and PET, are applied to diagnose and monitor progressive disease for MB.

The next-generation sequencing (NGS) has been applied to sequence circulating tumor DNA (ctDNA) from blood, especially for identifying *EGFR* mutations in non-small cell lung cancer. However, blood–brain barrier impedes detection of ctDNA in blood of a patient with brain tumor^4^. Recently, accumulating evidence has supported that circulating cell-free nucleic acids including DNA (cfDNA) and RNA (cfRNA), and even proteins in cerebrospinal fluid (CSF) are potential to revolutionize diagnosis and clinical care for CNS tumors, especially for glioblastoma (GBM) and MB^5^. We aim to explore the feasibility of liquid biopsy using CSF to facilitate diagnosis and to predict prognosis in MB.

To decipher pathogenesis of MB and apply targeted therapy, it is necessary to explore genetic background in different types of samples, such as tumor tissue and CSF, based on NGS platform. Because in our previous clinical genetic test reports, few MB-associated alterations were detected in tumor tissue. Therefore, deep sequencing on ctDNA in CSF and formalin-fixed paraffin-embedded (FFPE) tissue was performed to obtain comprehensive genome profiling. We hypothesized that genetic alteration in CSF may serve as a complementary role to tissue for monitoring disease progression.

Circulating cell-free DNA (cfDNA) is present in body fluid of healthy individuals as well as patients with cancer, such as plasma, urine and CSF. Circulating tumor DNA (ctDNA) is thought to be shed into circulation by apoptotic and necrotic tumor cells in patients with cancer^6^. Thus detection of ctDNA is of great value for early diagnosis of malignancy. For detecting ctDNA in the blood, the content of ctDNA is lower at earlier stage, compared to relatively late-stage^4,7^. Additionally, the difference of fragments size in ctDNA and cfDNA was utilized for early diagnosis of malignancies^8^. Furthermore, high-grade (WHO grades III and IV) brain tumors were more likely to harbor detectable ctDNA in CSF than low-grade ones^9^. Therefore, ctDNA detection in CSF plays a predictive role in monitoring disease progression.

Here, we determined the presence or absence of cfDNA in 58 CSF samples. In order to decipher medulloblastoma-associated alterations, deep sequencing based on NGS platform was applied to examine CSF, tumor tissue and blood in patients with MB. Notably, positive cfDNA in CSF is associated with disease progression or metastasis. It was more likely to detect medulloblastoma-associated alterations in CSF rather than tumor tissue. Shared alterations between CSF and matched tumor tissues could be detected when sample collected in short time interval.

## Methods

### Patients and sample collection

The protocol was approved by the institutional review board of Research Ethics Committee at Beijing Shijitan Hospital. Written informed consent was obtained from their parents of MB patients. All experiments were carried out in accordance with relevant guidelines and regulations.

In this prospective study, 58 patients with MB were recruited from *Beijing Tiantan Hospital of Capital Medical University and Beijing Shijitan Hospital of Capital Medical University* between April 2019 and Dec 2019*.* For each patient, CSF was collected by lumbar puncture or at the time of surgery. Additionally, tumor tissue samples were collected from 4 patients on primary-care and 7 patients with relapse. The matched plasma was isolated from 5 patients with relapse and treated with adjuvant radiotherapy or chemotherapy. Furthermore, whole blood was obtained from each patient, serving as germline DNA control.

### Isolation of cfDNA from CSF and plasma

The median volume of collected CSF is 5 mL. Fresh CSF was stored at 4 °C, and centrifugated (at 4 °C, 1400 rpm, for 5 min) within 2–3 h after collection. Cellular pellet was discarded. The sterile centrifuge tube was pre-cooled at − 20 °C. CSF supernatant was transferred to a cryotubes and stored at − 80 °C. Immediately before use, CSF was thawed in a thermostat water bath at 37 °C. Then, cfDNA was extracted from CSF supernatant or plasma according to the manufacturer’s protocol using QIAamp Circulating Nucleic Acid Kit (catalog #55114; QIAGEN, Valencia, CA).

### Isolation of tumor/germline DNA

Isolation of genomic DNA from FFPE tumor tissue or matched whole blood leukocytes was performed using QIAamp DNA FFPE Tissue Kit (Qiagen, Hilden, Germany) and blood/cell culture DNA kit (Qiagen, Valencia, CA, USA), respectively. Concentration of isolated (cell-free and genomic) DNA was quantified with Qubit 2.0 Fluorometer using Qubit dsDNA HS Assay kit (Life Technologies, Carlsbad, California, US) according to the manufacture’s protocol. DNA was stored at − 20 °C until it was used.

### Analysis of cfDNA and ctDNA

The presence and absence of cfDNA was inferred according to isolated DNA fragment length distribution, instead of quantity. To verify isolated cfDNA, the size of cfDNA fragment was assessed using Agilent 2100 Bioanalyzer. If peak appeared at 150–200 base pair (bp), it is considered as cfDNA, otherwise (> 1500 bp) as CSF cellular genomic DNA. And NGS library construction was not performed against CSF cellular genomic DNA. The detection method cannot determine whether cfDNA is derived from tumor tissue. Therefore, we adopted the term “cfDNA”. The term “ctDNA” was used only when tumor-associated mutations were detected in CSF or plasma^10^.

### DNA library construction and NGS sequencing

First, tumor genomic DNA was fragmented into 150–200 bp fragments with Bioruptor Pico. Libraries of fragmented DNA and cfDNA were constructed with KAPA Library Amplification Kit (KK2611/KK2612) according to standard protocols. Next, cfDNA libraries were captured with a designed 500-gene or 952-gene panel (Agilent), which contained the majority of brain tumor related genes. The genomic DNA libraries were applied with whole exome sequencing (WES). The captured samples were subjected to paired-end sequencing on the Illumina NovaSeq 6000 platform. Finally, the low quality reads were removed based on bioinformatics; while the high quality reads were mapped on human reference sequence (hg19) (Human Genome version 19) using tool Burrows–Wheeler Aligner software (BWA)^11^. The SNVs and In/Dels were detected using Genome Analysis Toolkit (GATK)^12^ and VarScan2^13^ and filtered out by dbSNP and 1000 Genome datasets. We selected at least 3 mutated reads with quality scores of > 30. In this study, we focused on mutations located in exons, such as nonsynonymous, synonymous, frameshift, non-frameshift and splicing sites. All the genes tested were verified with Integrative Genomics Viewer (IGV).

### Statistical analysis

Associations between CSF-cfDNA and clinical characteristics including age and quantity of DNA in CSF were evaluated by Mann–Whitney U tests. Clinical features such as recurrence, metastasis, tumor progression and quantity of DNA in CSF were compared between positive and negative CSF-cfDNA groups with Fisher’s exact tests or chi-square tests. All statistical tests were two-sided with a *p* ≤ 0.05 defined as statistical significance.

## Results

### The basic characteristics of patients

The patient population (64% male and 36% female) included infants, children or teenager, aged at 2–15 years old (median: 7). Demographic and clinical features were summarized in Table 1, including age, gender, medial histology, primary tumor location, initial diagnosis (M stage, molecular subgroup), recurrence, progression, with or without detectable CSF-cfDNA, and quantity of DNA in CSF. Tumors were located in the 4th ventricle in the majority of patients (n = 48). 7 patients had spinal spread, 3 had spinal solitary metastasis, 2 had craniospinal (cerebrospinal; cerebellum and spinal axis) spread and one had subventricular spread. Other tumors were located in vermis (n = 5), cerebellar hemisphere (n = 4) or cerebellopontine angle (CPA) (n = 1). Totally, 15 patients developed distant metastasis or local spread. Disease recurrence occurred in 7 patients. Among 58 patients, 15 had detectable cfDNA in CSF (as analyzed by Agilent 2100 Bioanalyzer). Eight patients developed progressive disease (PD) or died of PD. One patient exhibited partial remission (PR) after chemotherapy and surgery. The rest of patients achieved stable disease (SD) (n = 3) or complete remission (CR) (n = 46) after surgery, nevertheless, the patient 192D0463 died from radiation (RT) complication.

**Table 1:** Clinical characteristics of 58 patients with medulloblastoma.

Sample	Age (y)	Sex	Primary location	Histology	M stage	Molecular subgroup	Recurrence	CSF-cfDNA	DNA in CSF (ng)	Tumor progression
192D0695	5	M	4th ventricle	DMB	M0	SHH	No	Positive	14.7	CR
192D0640	7	M	4th ventricle	CMB	M0	Group 4	No	Positive	1995	CR
192D0394	6	M	4th ventricle	DMB	M0	SHH	No	Positive	17.99	CR
192D0360	3	F	4th ventricle and spinal spread	CMB	M3	Group4	No	Positive	84.8	PD, die
192D0593	7	M	4th ventricle and spinal spread	CMB	M3	Group 4	No	Positive	17.29	SD
192D0630	6	M	4th ventricle and spinal spread	CMB	M3	Group4	No	Positive	12.67	PR
192D0479	9	M	4th ventricle with spinal solitary metastasis	CMB	M3	Group4	No	Positive	12.88	CR
202D0009	3	M	4th ventricle and spinal spread	CMB	M3	Group 4	No	Positive	784	Die
202D0037	7	M	4th ventricle	CMB	M1	Group 4	No	Positive	90.4	CR
192D0406	8	F	Cerebellar hemisphere	CMB	M1	Group 3	No	Positive	28	CR
192D0582	5	F	Vermis	DMB	M0	SHH	Yes	Positive	24.22	PD
192D0584	3	M	4th ventricle with craniospinal spread	LMB	M3	SHH/Group 4	Yes	Positive	106.4	PD, die
192D0629	5	M	4th ventricle	CMB	M0	/	Yes	Positive	173.6	PD
192D0653	9	F	4th ventricle	CMB	M0	Group 4	Yes	Positive	777	PD, die
192D0674	10	M	4th ventricle and spinal spread	CMB	M3	Group 4	Yes	Positive	40.25	SD
192D0583	2	M	Cerebellar hemisphere	CMB	M0	SHH	Yes	Not detected	11.13	CR
192D0641	13	F	4th ventricle	CMB	M0	SHH	Yes	Not detected	28.42	PD
192D0663	2	M	4th ventricle	CMB	M0	Group 3	No	Not detected	9.8	PD
192D0463	6	M	4th ventricle	CMB	M0	Group 4	No	Not detected	6.8	Die from RT complication
202D0024	7	M	4th ventricle with spinal solitary metastasis	MEBN	M3	SHH	No	Not detected	5.76	SD
192D0408	12	M	4th ventricle and spinal spread	DMB	M3	SHH	No	Not detected	30.03	CR
202D0005	7	M	4th ventricle with spinal solitary metastasis	DMB	M3	SHH	No	Not detected	8.12	CR
192D0331	7	F	4th ventricle and subventricular spread	DMB	M2	SHH	No	Not detected	5.48	CR
192D0604	2	F	4th ventricle with craniospinal spread	CMB	M3	Group 4	No	Not detected	9.8	CR
192D0669	8	M	4th ventricle and spinal spread	CMB	M3	Group 4	No	Not detected	7.42	CR
192D0657	11	M	4th ventricle	DMB	M0	SHH	No	Not detected	77	CR
192D0362	5	F	4th ventricle	DMB	M0	WNT	No	Not detected	9.92	CR
192D0448	5	M	4th ventricle	CMB	M0	Group4	No	Not detected	6.88	CR
192D0449	5	M	4th ventricle	DMB	M0	Group3	No	Not detected	9.59	CR
192D0480	9	M	4th ventricle	CMB	M0	WNT	No	Not detected	8.4	CR
192D0532	11	M	4th ventricle	CMB	M0	Group4	No	Not detected	8.2	CR
192D0542	8	F	4th ventricle	CMB	M0	WNT	No	Not detected	7.6	CR
192D0591	13	M	4th ventricle	CMB	M0	Group4	No	Not detected	12.74	CR
192D0627	6	F	4th ventricle	CMB	M0	WNT	No	Not detected	8.4	CR
192D0571	4	M	4th ventricle	CMB	M0	Group3	No	Not detected	7.63	CR
192D0574	5	M	4th ventricle	CMB	M0	Group4	No	Not detected	12.39	CR
192D0585	8	F	4th ventricle	CMB	M0	WNT	No	Not detected	7.91	CR
192D0594	8	M	4th ventricle	CMB	M0	Group4	No	Not detected	12.95	CR
192D0631	3	M	4th ventricle	DMB	M0	Group4	No	Not detected	5.39	CR
192D0646	8	F	4th ventricle	CMB	M0	Group4	No	Not detected	8.26	CR
192D0652	15	M	4th ventricle	DMB	M0	SHH	No	Not detected	3.78	CR
192D0655	8	M	4th ventricle	CMB	M0	/	No	Not detected	8.19	CR
192D0662	2	F	4th ventricle	CMB	M0	Group3	No	Not detected	6.3	CR
192D0671	5	M	4th ventricle	DMB	M0	SHH	No	Not detected	3.5	CR
192D0696	2	M	4th ventricle	CMB	M0	Group4	No	Not detected	11.9	CR
202D0004	6	F	4th ventricle	DMB	M0	Group3	No	Not detected	8.54	CR
202D0007	11	F	4th ventricle	CMB	M0	WNT	No	Not detected	15.12	CR
202D0013	2	F	4th ventricle	DMB	M0	SHH	No	Not detected	3.71	CR
192D0590	8	M	4th ventricle	DMB	M0	SHH	No	Not detected	8.05	CR
202D0020	8	M	4th ventricle	CMB	M0	Group4	No	Not detected	6.3	CR
202D0029	7	F	4th ventricle	CMB	M0	WNT	No	Not detected	6.48	CR
192D0407	3	F	CPA	DMB	M0	SHH	No	Not detected	7.49	CR
192D0361	6	M	Cerebellar hemisphere	CMB	M0	SHH	No	Not detected	6.02	CR
192D0425	5	M	Cerebellar hemisphere	L/AMB	M0	SHH	No	Not detected	6.51	CR
192D0484	8	F	Vermis	CMB	M0	WNT	No	Not detected	10.16	CR
192D0592	12	F	Vermis	CMB	M0	Group4	No	Not detected	7.7	CR
192D0626	9	M	Vermis	CMB	M0	Group4	No	Not detected	7.14	CR
202D0032	8	F	Vermis	CMB	M0	Group4	No	Not detected	6.08	CR

CR, complete remission; PD, progressive disease; SD, stable disease; PR, partial remission; CPA, cerebellopontine angle.

### Relationship between CSF-cfDNA and clinical characteristics

In our patients with MB, CSF-cfDNA was not associated with age (*p* = 0.50, Mann–Whitney U test) or gender (*p* = 0.37, chi-square test). However, the presence or absence of cfDNA in CSF was associated with several clinical features, including recurrence (*p* = 0.0098, Fisher’s exact test), metastasis (*p* = 0.001, Fisher’s exact test), tumor progression (*p* = 0.003, Fisher’s exact test), as well as quantity of DNA in CSF (*p* < 0.001, Mann–Whitney U test) (Table 2). The median quantity of DNA in CSF with cfDNA (279 ng) is higher than that without cfDNA (11 ng).

**Table 2:** Associations between clinical characteristics and CSF-cfDNA.

Characteristic	All patients (n = 58)	With cfDNA (n = 15)	Without cfDNA (n = 43)	*p* value
Age (y); median (range)	7 (2–15)	6 (3–10)	7 (2–15)	0.50
**Sex**				0.37
Male; n (%)	37 (64)	11 (73)	26 (60)	
Female; n (%)	21 (36)	4 (27)	17 (40)	
**Recurrent, n (%)**				0.0098
No	51 (88)	10 (67)	41 (95)	
Yes	7 (12)	5 (33)	2 (5)	
**M stage, n (%)**				0.001
M0	43 (74)	6 (40)	37 (86)	
M1–3	15 (26)	9 (60)	6 (14)	
**Tumor progression**				0.003
CR or SD or PR	50 (86)	9 (60)	41 (95)	
PD or Dead	8 (14)	6 (40)	2 (5)	
**Quantity of DNA in CSF (ng)**				< 0.001
Median (range)	80 (4–1995)	279 (13–1995)	11 (4–77)	

### Medulloblastoma-associated alteration detected in CSF, tumor and plasma

**Figure 1:**
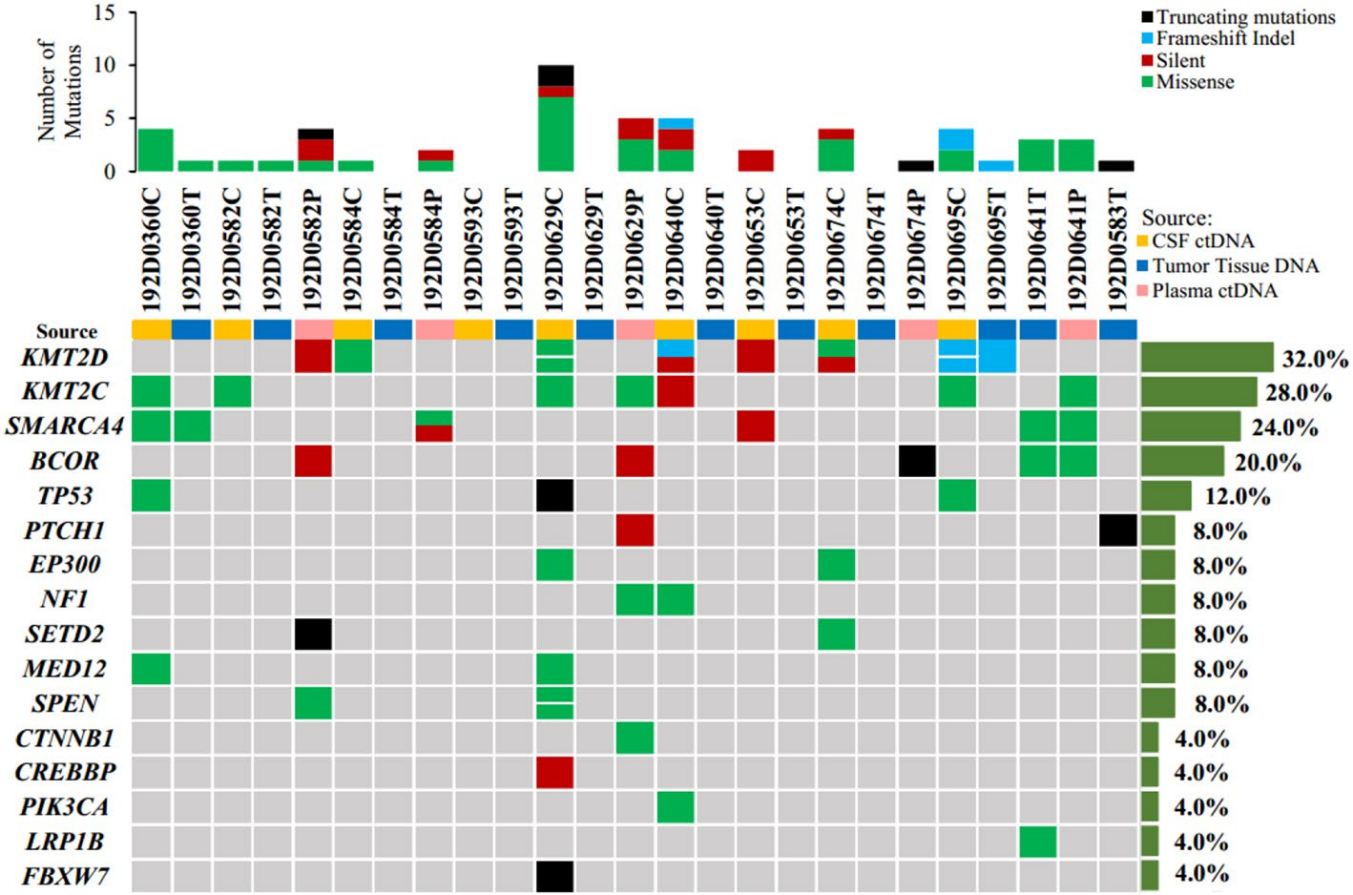
The genomic landscape of 11 medulloblastoma patients. Top, frequency of mutations per case; Bottom left, genetic alterations detected in 16 medulloblastoma-associated genes; Bottom right, frequency of mutations per gene in three different types of samples. C, CSF supernatant; T, tumor tissue; P, plasma.

For this study, eleven patients with MB were selected for genomic analysis. Seven patients developed recurrent diseases. Tissue DNA, CSF or plasma cfDNA from 6 patients were subjected to sequencing on NGS platform. The isolated tumor and germline DNA samples were sequenced with WES. The cfDNA isolation yielded from CSF and plasma was sequenced on NGS platforms with 500-gene panel, except for 192D0360 with 952-gene panel. Because of cancer genes were detected in those patients’ CSF or plasma, the term “cfDNA” be called “ctDNA” in following text^10^. Medulloblastoma-associated alterations detected in ctDNA from CSF were more abundantly present than genomic DNA from tumor tissue (Fig. 1). The most common genetic alterations were *KMT2D* (32.0%), *KMT2C* (28.0%), *SMARCA4* (24.0%), *BCOR* (20.0%), *TP53* (12.0%), *PTCH1* (8%), *EP300* (8%), *NF1* (8%), *SETD2* (8%), *MED12* (8%) and *SPEN* (8%). Notably, *BCOR* alterations were the most frequent in ctDNA from plasma. The presence of medulloblastoma-associated mutations in plasma may be due to the fact that patients with recurrent disease had been treated with adjuvant radiotherapy or chemotherapy. It was indicated that blood-brain barrier system could be damaged or destroyed in response to radiotherapy or chemotherapy^14–17^. It is surprising that medulloblastoma-associated somatic mutations from tumor tissues were detected in only 36% (4/11) of patients. We identified nonsynonymous SNVs in tumor tissues, such as *SMARCA4* (in 192D0360T and 192D0641T), *BCOR* and *LRP1B* in 192D0641T, as well as frameshift mutations of *KMT2D* in 192D0695T and truncating mutations of *PTCH1* in 192D0583T. Although it is possible that the low concordance between samples from the same individual could be resulted from the number of different detected alterations, the common medulloblastoma-associated alterations in tumor tissue were few in samples with more genes detected, such as 192D0583T, 192D0629T and 192D0674T (Fig. 1 and Fig. S1). Additionally, shared alterations between tissue and CSF or plasma were only found in case 192D0360, 192D0695 and 192D0641 (Fig. S1). It was not an obvious trend indicating that the increasing concordance of alterations between samples changed along with improvement of detectable genes.

### Shared genetic alterations between ctDNA from CSF and genomic DNA from tumor tissue

NGS-based detection of alterations was performed against nine paired tumor DNA and CSF-ctDNA to explore shared genetic mutations between CSF and tumor tissue. As shown in Table 3, shared alterations were detected in 2 out of 9 patients (22%). Two nonsynonymous SNVs in *SMARCA4* and *SETD8* genes and 2 synonymous SNVs in *DROSHA* and *MLLT3* genes were identified in case 192D0360. Frameshift mutation in *KMT2D* and nonsynonymous SNVs in *SNCAIP* were identified in case 192D0695. The most common alterations included truncating mutations in *KMT2D*^18^ and *SNCAIP* tandem duplication (somatic mutation), excluding from dominated somatic copy number aberrations (SCNA) in MB Group 4^19^. Nevertheless, no shared alterations were found in the remaining seven patients (78%).

**Table 3:** The shared alterations detected from CSF and tumor tissue in 9 cases.

Sample	Chromosome	Mutant functional type	Gene	Transcript	Location	cDNA Mutation	AA Mutation	MAF (%)
192D0360C	chr5: 31409426	synonymous SNV	*DROSHA*	NM_013235	exon31	c.3681G > A	p.A1227A	46.60
192D0360T	chr5: 31409426	synonymous SNV	*DROSHA*	NM_013235	exon31	c.3681G > A	p.A1227A	48.30
192D0360C	chr9: 20414280	synonymous SNV	*MLLT3*	NM_004529	exon5	c.564C > T	p.S188S	2.00
192D0360T	chr9: 20414280	synonymous SNV	*MLLT3*	NM_004529	exon5	c.564C > T	p.S188S	3.80
192D0360C	chr19: 11132513	nonsynonymous SNV	*SMARCA4*	NM_003072	exon19	c.2729C > T	p.T910M	40.90
192D0360T	chr19: 11132513	nonsynonymous SNV	*SMARCA4*	NM_003072	exon19	c.2729C > T	p.T910M	6.00
192D0360C	chr12: 123875223	nonsynonymous SNV	*SETD8*	NM_020382	exon3	c.179C > T	p.P60L	2.30
192D0360T	chr12: 123875223	nonsynonymous SNV	*SETD8*	NM_020382	exon3	c.179C > T	p.P60L	3.20
192D0695C	chr5: 121786553	nonsynonymous SNV	*SNCAIP*	NM_005460	exon10	c.2011A > T	p.M671L	4.80
192D0695T	chr5: 121786553	nonsynonymous SNV	*SNCAIP*	NM_005460	exon10	c.2011A > T	p.M671L	8.00
192D0695C	chr12: 49427079	frameshift insertion	*KMT2D*	NM_003482	exon39	c.11408_11409insTGGG	p.G3803fs	14.20
192D0695T	chr12: 49427079	frameshift insertion	*KMT2D*	NM_003482	exon39	c.11408_11409insTGGG	p.G3803fs	13.70

C, CSF supernatant; T, Tumor tissue; MAF, Mutant allele fraction; AA, Amino acid.

To clarify the reason about the detectable shared mutations, we then collected clinical information of the above nine patients (Table 4). We found that CSF collected by lumber puncture was only one month after operation for the case 192D0360 and 192D0593. The CSF from the case 192D0695 and 192D0640 was collected during operation. Shared alterations were found only in the case 192D0360 and 192D0695. The remaining CSF samples were collected at the time of recurrence, and mean time interval from the initial surgery is 15.7 months (range, 5.5–23 months). No shared alterations could be detected in those cases (Table 4). We guess that shared gene alterations might be detected only when the collecting time interval between CSF and matching tumor tissue was close.

**Table 4:** The time interval for sample collection between CSF and tumor tissue.

Sample	Gender	Onset age	Treatment stage	Collecting time interval between CSF and tumor	Shared alterations between CSF and tissue
192D0629	M	5	Recurrence after surgery	29 months after operation	N
192D0674	M	10	Recurrence after surgery	23 months after operation	N
192D0584	M	3	Recurrence after surgery	12 months after operation	N
192D0582	F	5	Recurrence after surgery	9 months after operation	N
192D0653	F	9	Recurrence after surgery	5.5 months after operation	N
192D0593	M	7	Initial therapy	1 month after operation	N
192D0360	F	3	Initial therapy	1 month after operation	Y
192D0695	M	5	Initial therapy	During operation	Y
192D0640	M	7	Initial therapy	During operation	N

Y, with shared alterations; N, without shared alterations.

## Discussion

It is challenging to monitor disease progression for patients with CNS tumors since repeated surgical resection is unfeasible, while the pathology diagnosis that distinguish proliferative/aggressive tumor, pseudoprogression or necrosis directly influenced treatment decision-making process^20^. Therefore, detection of tumor-derived cfDNA in CSF has become a minimally invasive approach to dynamically monitor tumor progression, which can facilitate targeted therapy for cancer patients.

In our study, cfDNA from 43 out of 58 CSF samples could not be detected. The presence of tumor-derived cfDNA from CSF was significantly related to primary location of tumor adjacent to CSF reservoir. The CNS neoplasms located in brain or spinal cord, where lesions adjacent to CSF reservoir were much more likely to harbor tumor-derived cfDNA in CSF^9^. In our patient population, tumor lesions interacted with CSF reservoir directly. The difference may be also caused by limitation of current detection techniques using CSF as liquid biopsy in childhood MB. Notably, CSF-cfDNA was undetected in the vast majority of patients who exhibited CR after surgery (without metastasis). cfDNA in CSF was detected in 15 patients. Among these 15 patients, five developed postoperative recurrence and PD, and 7 with initial therapy had varying degrees of metastasis (M1 or M3) or PD and even died of rapid PD. Only 3 patients achieved CR (without metastasis) after surgery in several months (range from 2 to 6 months). Furthermore, clinical stage, such as recurrence, metastasis and PD between positive and negative CSF-cfDNA was different statistically. Similar to previous studies, the levels of tumor-derived cfDNA in CSF reflected response to therapy or PD^21^. Additionally, the quantity of plasma-ctDNA was correlated with tumor burden, as a predictor for recurrence and progression^10^.

In our study, more medulloblastoma-associated gene mutations were identified in CSF than in tumor tissue (Fig. 1). This difference may be caused by sampling bias inherent in traditional biopsy and tumor heterogeneity. However, more tumor-derived genetic alterations were identified from CSF than tumor tissue, as important factors for pathogenesis of MB. Medulloblastoma-associated alterations were not detected in the majority of FFPE tumor tissues (Fig. 1). Similarly, genetic alterations in CSF-ctDNA were unable to be consistently detected for supratentorial brain tumors^22^. From another perspective, the mean sequencing depth for liquid biopsy (2000×) is higher than tumor tissue (110×), so that more alterations may be captured. Therefore, more reasonable sequencing scheme should be designed to verify our findings. Additionally, medulloblastoma-associated alterations were detected in blood, possibly attributed to adjuvant radiotherapy or chemotherapy for patients with postoperative recurrence. We may infer that brain tumor-related gene mutations are detectable in the blood of patients with MB who have been treated with radiotherapy or chemotherapy. However, since we did not collect blood samples from patients who were not treated with chemoradiotherapy, it remained unclear if mutation detection in ctDNA from plasma is applicable to all kinds of treated patients with brain tumors.

No or few medulloblastoma-associated alterations were detected from tumor tissue. Shared alterations were identified between CSF and matched tumor tissue in only 2 out of 9 patients. Shared missense mutations may contribute to pathogenesis of MB. For example, *SMARCA4* forms a protein complex with *CTNNB1*, which remodels chromatin and participates in WNT pathway^23^. *SETD8* (also known as *KMT5A*) regulates *TP53* activity and expression^24^. *KMT2D* (also known as *MLL2*) acts as a universal oncogenic driver to regulate epigenetic alterations. *SNCAIP* encodes synphilin-1, by binding to α-synuclein, which promotes the formation of Lewy bodies in the brains of patients with Parkinson’s disease^25,26^. Interestingly, in both patients, time interval for sample collection between CSF and tumor tissue was very short. CSF was collected one month after operation for 192D0360 whereas during operation for 192D0695. By contrast, CSF was collected during operation for 192D0640 whereas one month after surgery for 192D0593, but no shared alterations were detected from these 2 cases. CSF was obtained from the remaining 5 samples after disease recurrence. It may be easier to detect shared mutations between CSF and matched tumor tissue when time interval for sample collection is relatively short. CSF-ctDNA from patients with MB provides a comprehensive and genetically representative genome profiling only at the time of CSF collection. Finally, this result is consistent with previous findings that genomic evolution of brain tumors can change significantly over time^27–32^.

In conclusion, detectable cfDNA in CSF may predict disease progression. Deep sequencing on CSF-ctDNA derived from patients with MB may obtain more tumor-specific mutations than matched tissue. This finding may help targeted therapy and drug development. Furthermore, CSF samples collected at different time points could represent snap-shot genome landscape only at the time of acquisition. Our finding may help diagnosis and monitoring progressive disease for patients with MB. This research is still ongoing. Thus, prospective studies are needed to confirm if CSF-ctDNA detection as liquid biopsy could be used in routine clinical therapy for MB, and if CSF-ctDNA gene mutations could guide targeted therapy in recurrent childhood MB.

## Supplementary Information

Supplementary Information

## References

[1] Fiaschetti G (2014). NOTCH ligands JAG1 and JAG2 as critical pro-survival factors in childhood medulloblastoma. Acta Neuropathol. Commun..

[2] Louis DN (2016). The 2016 World Health Organization classification of tumors of the central nervous system: A summary. Acta Neuropathol..

[3] Cefalo G (2014). Temozolomide is an active agent in children with recurrent medulloblastoma/primitive neuroectodermal tumor: An Italian multi-institutional phase II trial. Neuro Oncol..

[4] Bettegowda C (2014). Detection of circulating tumor DNA in early- and late-stage human malignancies. Sci. Transl. Med..

[5] Mattox AK, Yan H, Bettegowda C (2019). The potential of cerebrospinal fluid–based liquid biopsy approaches in CNS tumors. Neuro Oncol..

[6] Snyder MW, Kircher M, Hill AJ (2016). Cell-free DNA comprises an in vivo nucleosome footprint that informs its tissues-of-origin. Cell.

[7] Newman AM (2014). An ultrasensitive method for quantitating circulating tumor DNA with broad patient coverage. Nat. Med..

[8] Mouliere F (2018). Enhanced detection of circulating tumor DNA by fragment size analysis. Sci. Transl. Med..

[9] Wang Y (2015). Detection of tumor-derived DNA in cerebrospinal fluid of patients with primary tumors of the brain and spinal cord. Proc. Natl. Acad. Sci..

[10] Wu X (2020). Circulating tumor DNA as an emerging liquid biopsy biomarker for early diagnosis and therapeutic monitoring in hepatocellular carcinoma. Int. J. Biol. Sci..

[11] Li H, Durbin R (2009). Fast and accurate short read alignment with Burrows–Wheeler transform. Bioinformatics.

[12] DePristo MA (2011). A framework for variation discovery and genotyping using next-generation DNA sequencing data. Nat. Genet..

[13] Koboldt DC (2012). VarScan 2: Somatic mutation and copy number alteration discovery in cancer by exome sequencing. Genome Res..

[14] Stewart DJ (1994). A critique of the role of the blood-brain barrier in the chemotherapy of human brain tumors. J. Neurooncol..

[15] Van Vulpen M, Kal HB, Taphoorn MJ, El-Sharouni SY (2002). Changes in blood-brain barrier permeability induced by radiotherapy: Implications for timing of chemotherapy? (Review). Oncol. Rep..

[16] Hall WA (2006). Osmotic blood-brain barrier disruption chemotherapy for diffuse pontine gliomas. J. Neurooncol..

[17] Fauquette W, Amourette C, Dehouck MP, Diserbo M (2012). Radiation-induced blood-brain barrier damages: an in vitro study. Brain Res..

[18] Skowron P, Ramaswamy V, Taylor MD (2015). Genetic and molecular alterations across medulloblastoma subgroups. J. Mol. Med. (Berl.).

[19] Northcott PA (2012). Subgroup-specific structural variation across 1000 medulloblastoma genomes. Nature.

[20] Woodworth GF (2013). Histopathological correlates with survival in reoperated glioblastomas. J. Neurooncol..

[21] Momtaz P (2016). Quantification of tumor-derived cell free DNA(cfDNA) by digital PCR (DigPCR) in cerebrospinal fluid of patients with BRAFV600 mutated malignancies. Oncotarget.

[22] Pan C (2019). Molecular profiling of tumors of the brainstem by sequencing of CSF-derived circulating tumor DNA. Acta Neuropathol..

[23] Robinson G (2012). Novel mutations target distinct subgroups of medulloblastoma. Nature.

[24] Shi X (2007). Modulation of p53 function by SET8-mediated methylation at lysine 382. Mol. Cell.

[25] Engelender S (1999). Synphilin-1 associates with alpha-synuclein and promotes the formation of cytosolic inclusions. Nat. Genet..

[26] Chung KK (2001). Parkin ubiquitinates the alpha-synuclein-interacting protein, synphilin-1: Implications for Lewy-body formation in Parkinson disease. Nat. Med..

[27] Johnson BE (2014). Mutational analysis reveals the origin and therapy-driven evolution of recurrent glioma. Science.

[28] Kim H (2015). Whole-genome and multisector exome sequencing of primary and post-treatment glioblastoma reveals patterns of tumor evolution. Genome Res..

[29] Kim J (2015). Spatiotemporal evolution of the primary glioblastoma genome. Cancer Cell.

[30] Wang J (2016). Clonal evolution of glioblastoma under therapy. Nat. Genet..

[31] Aihara K (2017). Genetic and epigenetic stability of oligodendrogliomas at recurrence. Acta Neuropathol. Commun..

[32] Miller AM (2019). Tracking tumour evolution in glioma through liquid biopsies of cerebrospinal fluid. Nature.

