# Supplementary material for: Exploring genetic alterations in circulating tumor DNA from cerebrospinal fluid of pediatric medulloblastoma
Source: Sci Rep. 2021 Mar 11;11:5638. doi: 10.1038/s41598-021-85178-6 (PMC7952732; doi:10.1038/s41598-021-85178-6)
Supplement: Supplementary file 1 — Supplementary Information [file 41598_2021_85178_MOESM1_ESM.docx]

**Exploring genetic alterations in circulating tumor DNA from cerebrospinal fluid of pediatric medulloblastoma**

Yanling Sun^1^, Miao Li^1^, Siqi Ren^1^, Yan Liu^1^, Jin Zhang^1^, Shuting Li^1^, Wenchao Gao^1^, Xiaojun Gong^1^, Jingjing Liu^1^, Yuan Wang^1^, Shuxu Du^1^, Liming Sun^1^, Wanshui Wu^1^&Yongji Tian^2^


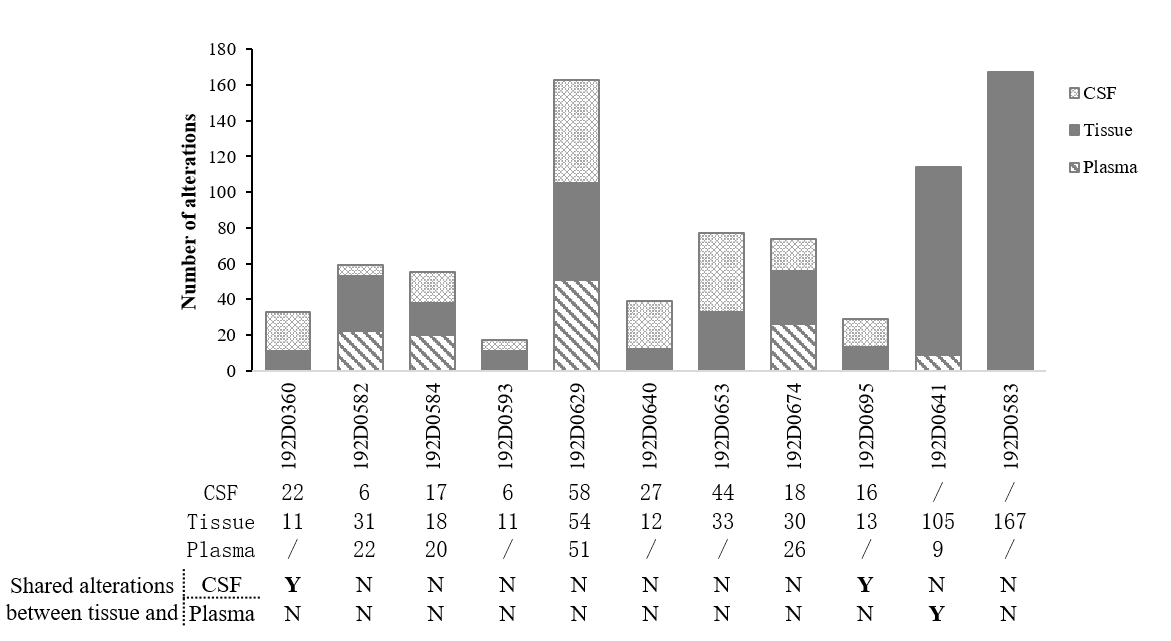


**Supplemental Figure 1. Total number of alterations in CSF, tissue and plasma of medulloblastoma.** Symbol “ / ”, the samples were not acquired; Y, with shared alterations; N, without shared alterations.
